# A Non-Native Prey Mediates the Effects of a Shared Predator on an Ecosystem Service

**DOI:** 10.1371/journal.pone.0093969

**Published:** 2014-04-09

**Authors:** James E. Byers, Rachel S. Smith, Heidi W. Weiskel, Charles Y. Robertson

**Affiliations:** 1 Odum School of Ecology, University of Georgia, Athens, Georgia, United States of America; 2 Skidaway Institute of Oceanography, Savannah, Georgia, United States of America; Dauphin Island Sea Lab, United States of America

## Abstract

Non-native species can alter ecosystem functions performed by native species often by displacing influential native species. However, little is known about how ecosystem functions may be modified by trait-mediated indirect effects of non-native species. Oysters and other reef-associated filter feeders enhance water quality by controlling nutrients and contaminants in many estuarine environments. However, this ecosystem service may be mitigated by predation, competition, or other species interactions, especially when such interactions involve non-native species that share little evolutionary history. We assessed trophic and other interference effects on the critical ecosystem service of water filtration in mesocosm experiments. In single-species trials, typical field densities of oysters (*Crassostrea virginica*) reduced water-column chlorophyll *a* more strongly than clams (*Mercenaria mercenaria*). The non-native filter-feeding reef crab *Petrolisthes armatus* did not draw down chlorophyll *a*. In multi-species treatments, oysters and clams combined additively to influence chlorophyll *a* drawdown. *Petrolisthes* did not affect net filtration when added to the bivalve-only treatments. Addition of the predatory mud crab *Panopeus herbstii* did not influence oyster feeding rates, but it did stop chlorophyll *a* drawdown by clams. However, when *Petrolisthes* was also added in with the clams, the clams filtered at their previously unadulterated rates, possibly because *Petrolisthes* drew the focus of predators or habituated the clams to crab stimuli. In sum, oysters were the most influential filter feeder, and neither predators nor competitors interfered with their net effect on water-column chlorophyll. In contrast, clams filtered less, but were more sensitive to predators as well as a facilitative buffering effect of *Petrolisthes*, illustrating that non-native species can indirectly affect an ecosystem service by aiding the performance of a native species.

## Introduction

Although non-native species often negatively affect ecosystem services in their introduced ranges (e.g., [1]), they can also positively influence ecosystem services by complementing the roles of native service providers or mitigating their loss [2]–[6]. For example, Pattemore and Wilcove [5] show that an invasive rat and bird in New Zealand are able to partially replace pollination services where native pollinators have declined. Similarly, Mattingly et al. [6] show that an invasive plant in the southeastern USA has boosted local nitrogen fixation and is actually enhancing native tree growth. In many examples like these, the effect of the invader is to directly supply an ecosystem service that replaces or enhances the service of native species. However, regardless of whether the effects of the invader on ecosystem services are positive or negative, most studies focus exclusively on how the alteration of effects is produced through density-mediated changes that occur as a result of the invasion. It seems logical, but seldom specifically explored, that non-native species could also indirectly affect ecosystem services through alteration of species traits, for example by altering the performance of important native service providers. For many communities of native species, such trait-mediated indirect interactions have been found to be of equal or greater magnitude to direct, density-mediated effects [7], [8].

Understanding how effects on ecosystem services operate is increasingly important since non-native species now account for a sizable portion of biota in many habitats, including coastal environments [9]–[11]. Furthermore, with rising human influence on habitat and water quality of estuaries, there is a growing need to quantify the ecosystem services provided by coastal habitats (e.g. [12]–[14]). However, data on key ecosystem services are surprisingly rare, even for common habitats such as oyster reefs. In particular, we lack a basic understanding of how the magnitude and nature of ecosystem services may change spatially and temporally as a function of species interactions, especially those involving non-native species.

In this paper we consider the direct and indirect impacts of a non-native species on water filtration. Water filtration is a critical ecosystem service provided by oysters and oyster reef-dwelling species that is likely affected by many biological and environmental factors. Oyster reef construction using the native oyster *Crassostrea virginica* Gmelin 1791 is a major restorative technique used in many coastal areas along the eastern seaboard of the United States, especially in the southeast [15]. Reef construction is justified in part by its purported effect on enhancing water quality [16], [17], and by earlier lab studies that suggest that *C. virginica* is highly efficient at drawing down material out of the water column (e.g., [18], [19]). Several studies have extrapolated individual oyster feeding rates to approximate the time needed for the oysters to process the overlying water column [20]–[23]. However, these calculations vary substantially in their conclusions over whether oysters have a controlling influence on water quality. Few *in situ* or experimental data exist on how well oysters contribute to water clearance in realistic settings [24]–[28].

We advocate that examination of the most common filter feeding reef species in concert adds critical, community-level insight to our understanding of this ecosystem function. For example, it would be informative to know how oysters' filtration rates compare against other filter feeders in the environment, as well as how intervening variables in the environment, especially biological interactions, might modify projected rates. Interactions with non-native species may be particularly influential since such species lack a shared evolutionary history with the species in the native community [29], [30]. Along with the oysters themselves, oyster reefs in the southeastern US harbor a filter-feeding native clam and non-native crab species, both of which may influence overall reef filtration rates. In combination, these filter-feeding species could have inhibitory effects on net filtration if they interact negatively, or additive (or even synergistic) effects if they feed on complementary planktonic resources or otherwise influence other species' filtering behavior.

Filtration by species when alone and in combination may also be affected by the presence of predators. Predators, like the mudcrab *Panopeus herbstii* H. Milne Edwards 1834, are abundant on oyster reefs, and can reduce prey density through consumption, which reduces the prey's net effect on the ecosystem service of water filtration. Conversely, predators may have positive effects on filtration if they preferentially prey upon the inferior filter feeders and reduce competition and interference effects for those individuals that remain. Predators may also influence the prey's net effect through non-consumptive effects that alter the behavior or physiology of the prey in a way that reduces the prey's per capita feeding rates [31], [32]. Non-consumptive predatory influences have been increasingly shown to be important in estuarine environments in a number of these species [33]–[35].

We compared filtration rates of two dominant native bivalve filter feeders (*Crassostrea virginica* and *Mercenaria mercenaria* Linnaeus 1758) in Georgia to the invasive crab, *Petrolisthes armatus* Gibbes 1850 (hereafter *Petrolisthes*), a relatively new non-native species on the Georgia coast. This small, filter-feeding crab reaches enormous densities on reefs, sometimes more than a thousand per square meter [36], but can experience large swings in population size due to low temperature sensitivity [37]. This crab may compete or interfere with the bivalves' feeding rates, for example, if olfactory cues or tactile disturbance from the crabs cause periodic filtration cessation in the bivalves. But such interactions are hard to predict given the paucity of natural history information on the crab as well as its lack of evolutionary history with the other native filter feeders.

Here we use single and multiple species combinations to experimentally quantify possible competitive or synergistic effects on filtration rates among the oysters, clams, and *Petrolisthes*. We further examine how these filter feeders' performance and interactions with each other are affected by predator presence. Thus, with our approach we can address whether *Petrolisthes* compromises or enhances filtration rates and whether its effects operate directly or indirectly through interactions with the species' shared predator.

## Methods/Approach

### Overview

To compare filtration abilities we quantified chlorophyll *a* drawdown by three abundant oyster reef species: the oyster *Crassostrea virginica*, the hard clam *Mercenaria mercenaria*, and the invasive crab *Petrolisthes armatus*. Using a laboratory seawater system at the Skidaway Institute of Oceanography, we examined short-term (3-hour) filtration rates of the species in aquaria. Each species was measured alone, in all pairwise combinations, and all together to test whether filtration is altered by interspecific interactions. Finally, to assess whether base filtration characteristics are altered by the presence of a predator, we performed a second experiment in which we added a common predator of all three filter feeders (the mud crab, *Panopeus herbstii*) to each treatment.

### Study animals

We collected oysters and crabs at low tide from intertidal oyster reefs at Priest Landing, located in the Wilmington River estuary in southeast Georgia (31° 57′45.34″ N, 81°0′ 48.08″ W). To facilitate obtaining enough clams of a standardized size class, we purchased 15–20 mm *M. mercenaria* from Bay Shellfish Company, Palmetto, FL in June 2010, and grew them in the intertidal mudflat at Priest Landing until the start of laboratory experiments in November 2012, at which point *M. mercenaria* had a mean shell length of 52.6 mm. (The hatchery-born clams are from Atlantic coast brood stock that had ample wild clams continually mixed in to ensure genetic diversity). Following field collection, we cleaned the oysters and clams of epibionts, separated individual oysters from oyster clumps, and weighed and measured all individuals (Table 1). We used large adults of the two bivalve species that are mostly invulnerable to crab predation because we wanted to focus on non-consumptive predator effects on filtration rates. Because the study organisms used are all invertebrates, no specialized animal care protocol was required. Organisms were collected under a scientific collection permit from the Georgia Department of Natural Resources on public land accessed through boat landings at the Skidaway Institute of Oceanography.

**Table 1:** Length, wet whole organism weight, and dry tissue weight measurements and corresponding standard deviations (SD) for study animals.

Species	Sample size	Length (mm)	SD	Wet Weight (g)	SD	Dry Tissue Weight (g)	SD
Oyster	540	62.03	11.97	23.02	9.95	0.63	0.3
Clam	162	52.55	3.98	71.73	14.22	1.14	0.34
*Petrolisthes armatus*	50	6.4	1.45	0.3	0.23	-	-
*Panopeus herbstii*	28	25.96	1.9	-	-	-	-

Measurements for *Petrolisthes* and *Panopeus* are from a randomly sampled subset. Oyster and clam lengths were measured from umbo to the ventral margin; length measurement for crabs refers to carapace width. A “-” represents a measurement that was not pertinent for that species in our experiment.

We kept the animals in separate containers in a flow-through seawater system to acclimate them to laboratory conditions for at least 24 hours prior to each experiment. Seawater was pumped directly from the Wilmington River, and then passed through a series of gravel filters and a sand filter prior to entering the lab flow-through system. We collected 10 liters of water from the system and passed it through a 10 μm sieve to determine the zooplankton concentration, which we counted under a dissecting microscope and observed to be extremely low (1 copepod/10 L). During the experiments, laboratory water temperatures ranged between 14.6 and 16.3°C, which was comparable to natural field temperatures for this time of year [38].

### Experimental set-up

We wanted to measure collective chlorophyll *a* drawdown by multiple individuals. To standardize comparisons across species, we used equalized total wet mass of oysters and clams (54 oysters, 18 clams) in our treatments (Table 1). We chose a value for total mass that roughly equated to densities of oysters measured in field surveys (Byers, unpublished data). We used an equivalent clam mass to keep the treatments matched, and thus readily comparable against one another. This resulted in clam densities somewhat higher than typical field densities in this area [39]; however, high clam densities should make our results conservative since the results reflect very low comparative influence of clam filtration. For completeness we also performed a separate, complementary experiment to compare filtration between clams and oysters that standardized the treatments by dry tissue mass (54 oysters, 30 clams), but this approach made little difference to the results. Dry mass for clams was calculated by creating a size-dry mass regression from a sample of 10 clams from our experimental population. For oysters we used a standard published size-dry weight regression from Ross and Luckenbach [40] (dry weight = 0.00003 x shell height^2.4^; R^2^ = 0.80). We chose *Petrolisthes* densities to approximate the low end of the density range previously observed in surveys within the Wilmington River estuary (500/m^2^) [36] (Table 2).

**Table 2:** Experimental treatments, the total number of animals per treatment, and the average and standard deviation (SD) in the drawdown of chlorophyll *a* concentration (μg/L) over the three hour experimental period for each treatment.

		Total chlorophyll *a* drawdown (μg/L)			
		Predators Absent		Predators Present	
Treatment	Number of Organisms	Average	SD	Average	SD
Control	0	18.61	1.80	17.19	2.12
O	54	29.52	2.33	26.37	5.33
CL	18	23.43	2.19	17.38	4.77
P	50	13.69	2.46	18.06	2.16
O-CL	54 O+18 CL	36.33	7.87	32.35	7.36
O-P	54 O+50 P	26.30	7.35	24.95	4.50
CL-P	18 CL +50 P	22.00	7.70	20.83	0.61
O-CL-P	54 O+18 CL +50 P	37.01	5.84	36.29	3.27

Treatment labels correspond to: controls (with no macro-organisms); oyster *Crassostrea virginica* (O); clam *Mercenaria mercenaria* (CL); and crab *Petrolisthes armatus* (P). All treatments were run both with and without *Panopeus herbstii* predators and replicated four times each, except O and O-CL, which were replicated six times each.

### Chlorophyll a drawdown by filter feeders

We used an additive design that allowed us to observe differences in chlorophyll *a* drawdown between individual species, species in all pairwise combinations, and all three species together for a total of 8 treatments, including a no-species control (Table 2). We set each treatment in a separate, individually aerated, 18-L plastic tank (42.9 cm×29.2 cm×24.5 cm) with flow-through seawater and no sediment on the bottom. We did not use sediment because we did not want to risk sediment becoming suspended and interfering with fluorometry measurements or behavioral observations. We ran one replicate of each treatment in a randomized block and we ran four replicate blocks on successive days. In each block, we measured chlorophyll *a* drawdown over a 3-hour time period. We measured chlorophyll *a in vivo* fluorescence with a WET Labs FLNTUSB fluorometer, which was standardized against extracted chlorophyll *a* measured on a Turner 10AU fluorometer from water samples taken at the beginning, middle, and end of one trial, as well as from known serial dilutions of algal feed [41]. We attached the fluorometer to a PVC stand that held the fluorometer underwater at a constant, downward angle in the treatment containers.

At the beginning of each trial, we turned off the water flow into the first aerated tank to create a closed system. We chose zero net flow because it is tractable. It is also realistic, as there are periods of slack tide for upwards of an hour (out of a 6-hour high tide) at our collection sites. However, an active aeration stone was present in each tank to maintain some water movement. We added 5 ml of commercial algae commonly used in aquaculture operations (Instant Algae Shellfish Diet 1800, Reed Mariculture, Campbell, CA, USA). The algal cells in the Shellfish Diet are non-viable, but intact, and are a mix of four marine microalgae: *Isochrysis* (40%), *Pavlova* (15%), *Tetraselmis* (25%), *Thalassiosira weissflogii* (20%). The amount of algal diet addition was intended to approximate the highest chlorophyll *a* levels observed in the Skidaway River estuary, which reach up to 70 μg/L [38], [42]. Our average initial algal concentration was 45.7 μg/L (±6.3 SD). Starting with high chlorophyll *a* levels allowed us to enhance the contrast of drawdown by our treatments. The tank then sat undisturbed for two minutes, after which we immersed the fluorometer. Two chlorophyll *a* measurements were taken within two minutes; simultaneously, we turned off water flow in the second treatment, added 5 ml of algal feed, and allowed the second treatment to sit. After two minutes, we moved the fluorometer to the second treatment to take two chlorophyll *a* measurements in the subsequent two minutes. This process was repeated for the following six treatments, until algal additions were added to all treatments. We then moved the fluorometer sequentially among treatments every two minutes over the course of three hours, taking two chlorophyll *a* measurements with each progression. Trials were intentionally kept short to minimize accumulation of olfactory cues in our closed system. Although lab measurements on olfactory responses of these species have been shown to be consistent with data from field situations where flow and turbulence levels are moderate [35], we acknowledge that olfactory responses could be accentuated compared to field measurements.

### Predator addition experiment

Immediately following the above trials, we performed a second set of trials to assess the effects of predator presence on chlorophyll *a* drawdown of filter feeders. We conducted four replicate trials as described above, but with each algal addition at the beginning of the trial, one *P. herbstii* was also added to the treatment (including the control). Additionally, in order to have contemporaneous controls of non-predator effects on drawdown, two extra oyster and oyster-clam treatments without *P. herbstii* predators were included in these trials. These treatments did not differ significantly from their matching treatments in the non-predator trials, so the extra replicates of these two treatments were used in the non-predator analyses. We also added two extra replicates of the oyster and oyster-clam treatments with predators to this set of trials to balance the number of replicates of these two treatments between the predator and non-predator trials. We generally noted biotic responses including the feeding-related activities of the filter feeders (bivalve time spent gaping, crab particle feeding and activity), dead prey, and predator activity levels.

Our response variable was chlorophyll *a* drawdown, which we standardized in our treatments relative to the chlorophyll loss in the control treatment for each trial. The loss of chlorophyll *a* over three hours in controls was high (∼40%), but consistently similar in every trial (standard deviation  = 2.95). Losses in the controls were likely due primarily to settling. We explored light-triggered chlorophyll *a* degradation as a possible loss mechanism by running controls in a darkened refrigerator (4 C) and compared this loss to a control under ambient conditions. The rate of loss in fluorescence was nearly identical. Loss mechanisms such as bacterial or viral attack were considered but discounted because of the short time scales involved; moreover, the loss in fluorescence was not slowed appreciably at 4 C, which would have slowed degradation. Fluorescence data gathered in controls were subtracted from the total signal in the treatments concurrently run within a block.

### Data analysis

Often a logit transformation is needed for non-binomial proportion data [43]. However, in this case the data were more normal without transformation and residuals showed no evidence of nonlinearity or heteroscedasticity so we did not transform the response variable.

Several *a priori* statistical comparisons were planned. First we used ANOVA to compare chlorophyll *a* drawdown among the three single species treatments, followed by a post hoc Tukey test to differentiate specific treatment differences. Second, we used the same approach to compare among the oyster, clam, and combined oyster-clam treatments to test for additivity or inhibitory effects when both bivalves were combined. Third, we performed three pairwise t-tests (assuming unequal variances), testing each bivalve treatment with and without *Petrolisthes*, to test for possible interference from this crab species. Finally, to discern which filter-feeding species and combinations of species performed differently in the presence of predators, we performed all of these same analyses on the trials done in the presence of *P. herbstii* (Table 3). We also compared each treatment with predators to its analogue treatment without predators with t-tests. Statistical analyses were performed in JMP v.10.

**Table 3:** Statistical summary of treatment comparisons using ANOVAs, post-hoc Tukey tests, and t-tests assuming unequal variances.

*A priori* comparisons:	Predators Absent	Predators Present
O vs. CL vs. P	F = 60.2, df = 13, P<0.0001; Tukey: all groups different from each other	F = 23.3, df = 13, P<0.0001; Tukey: Oyster A, Clam B, *Petrolisthes* B
O vs. CL vs. O-CL	F = 37.2, df = 15, P<0.0001; Tukey: all groups different from each other	F = 14.8, df = 15, P = 0.0004; Tukey: Clam A, Oyster B, Oyster-Clam B
O vs. O-P	t = 0.23, df = 4.5, P = 0.83	t = -0.04, df = 7.5, P = 0.97
CL vs. CL-P	t = 0.20, df = 4.5, P = 0.85	t = 2.56, df = 5.97, P = 0.04
O-CL vs. O-CL-P	t = −0.46, df = 5.2, P = 0.67	t = 1.3, df = 7.8, P = 0.23

The response variable was relative chlorophyll *a* loss standardized to the controls. Data were normal and residuals vs. predictor variables showed no evidence of nonlinearity or heteroscedasticity. For Tukey tests the capital letters after the treatment names represent treatments that are significantly different.

## Results

### Chlorophyll a drawdown by filter feeders

**Figure 1:**
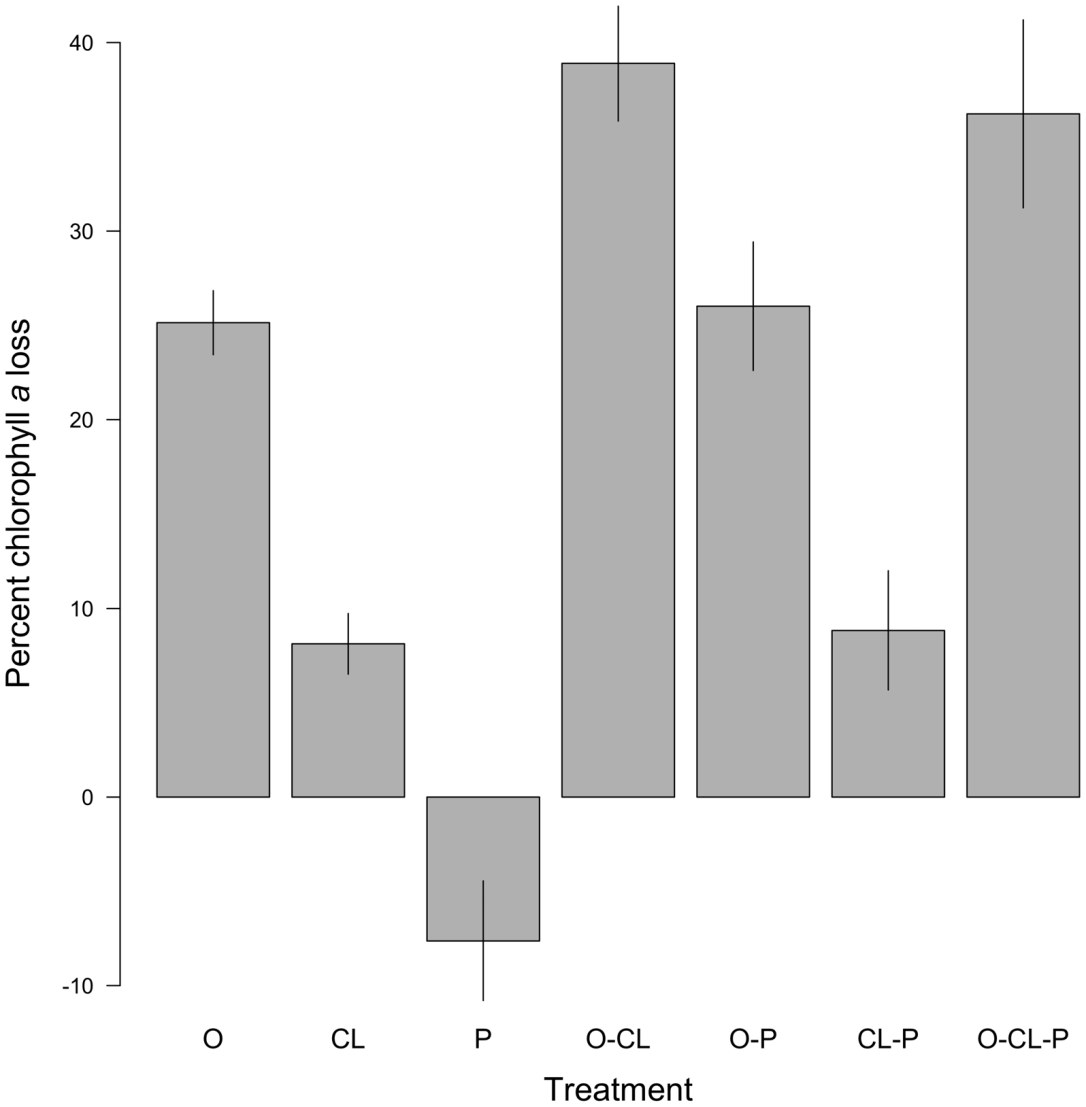
Mean percent chlorophyll *a* loss (± SE) by treatment over three hours, standardized by percent chlorophyll *a* loss in control treatment. Treatment labels correspond to oyster *Crassostrea virginica* (O); clam *Mercenaria mercenaria* (CL); crab *Petrolisthes armatus* (P). All treatments were significantly different from the controls (0%) in two-tailed t-tests (or 1-tailed t-tests as was the case for the *Petrolisthes* (P) and Clams-*Petrolisthes* (CL-P) treatments).

Chlorophyll *a* drawdown differed among the individual species (Tables 2 and 3), with oysters extracting significantly greater quantities of chlorophyll *a* compared to the clam and *Petrolisthes* treatments (Figure 1). The relationship between the oyster and the clam treatments was consistent in an additional trial set that compared species with population sizes determined by equivalent dry tissue weights, rather than total wet weights [Mean standardized percent chlorophyll *a* loss (± SD) over three hours: oysters 41.2±1.6; clams 4.1±3.3].

Chlorophyll *a* decreased in the control treatment, likely due to algal particle settlement. Treatments were standardized to the control to account for this ambient loss (Figure 1). Loss of chlorophyll *a* in the *Petrolisthes* treatment was slightly, but significantly less than the control, perhaps as a result of particle re-suspension by energetic crab movement (1-way t-test, t = −2.4, df = 3, P = 0.048). We observed *Petrolisthes* actively moving their mouth parts throughout the experiments; however, their filtering activity did not negatively affect chlorophyll *a* concentrations.

Multi-species treatments that allowed for observation of interspecific interactions showed that chlorophyll *a* drawdown of oysters and clams together was generally additive (Figure 1, Table 3). However, the presence of *Petrolisthes* did not significantly affect chlorophyll *a* drawdown of either oysters or clams, or the combined drawdown of the two bivalve species (Figure 1, Table 3). There was no evidence of the positive influence of *Petrolisthes* on chlorophyll *a* that was observed when it was alone.

### Predator addition experiment

#### Comparing analogous treatments between the no predator and predator addition trials

**Figure 2:**
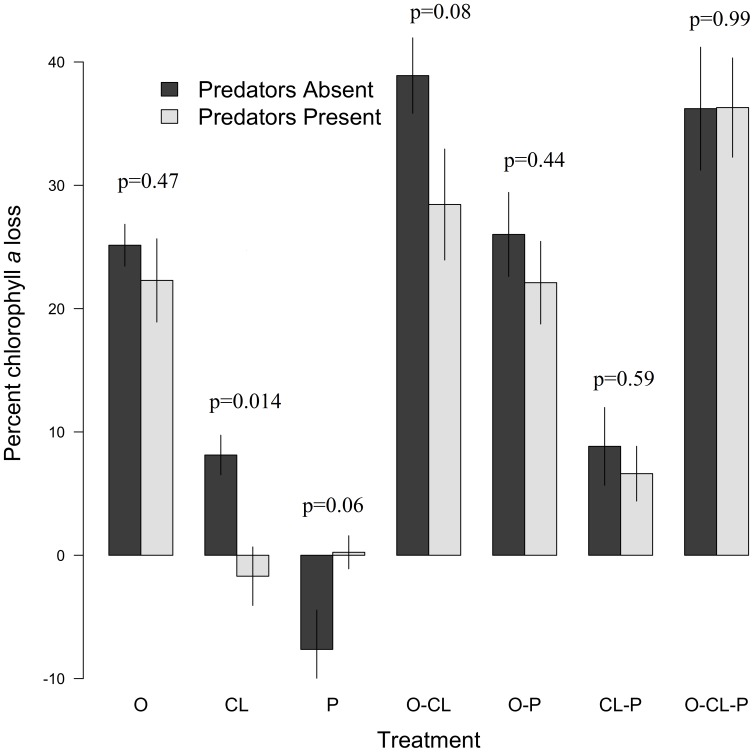
Standardized mean percent chlorophyll *a* loss (± SE) by treatment over three hours, in presence and absence of mud crab *P. herbstii* predators. Treatment codes are the same as in Figure 1. Data from Predators Absent trials are the same as those presented in Figure 1 but shown again here to ease comparison with the Predator Present trials. P values from comparison of each pair of bars (i.e. each treatment with and without predators) are shown.

Chlorophyll *a* drawdown by filter feeders decreased somewhat in the presence of *P. herbstii* (Figure 2). However, a reduction in chlorophyll *a* drawdown in the presence of crab predators was only significant in the clam treatment (Figure 2). Predators had some effect on chlorophyll *a* drawdown by *Petrolisthes* (p = 0.06); however, its drawdown actually increased to match the level of the control treatment, perhaps because predator presence limited *Petrolisthes* activity and re-suspension of algal particles. We observed that *Petrolisthes* ceased filtering activity in the presence of predators and, in some cases, were preyed upon, as evidenced by mangled bodies with missing parts. Chlorophyll *a* drawdown by the combined oyster and clam treatment decreased in the presence of predators, but not significantly. All three bivalve species treatments (oysters, clams, oysters and clams) containing *Petrolisthes* responded similarly with predators compared to their responses without predators (Figure 2).

#### Comparing single and multiple species trials within the predator addition trials

Clams drew down significantly less chlorophyll *a* than both the oyster and the oyster-clam treatments, which both responded the same (Table 3). The presence of *Petrolisthes* in the oyster treatment when a predator was added made no significant difference to chlorophyll *a* drawdown, but *Petrolisthes* presence did significantly boost chlorophyll *a* drawdown by clams 3.45 μg/L, or 20%, when a predator was present (Tables 2 and 3, Figure 2). The addition of *Petrolisthes* to the combined oyster and clam treatment also boosted drawdown by a similar amount in absolute terms (3.94 μg/L) (Table 2); however, this boost in relative terms was 12% and not quite statistically significant (Table 3, Figure 2).

## Discussion

The net effects of non-native species that severely reduce or displace native species on ecosystem services are often negative [44]–[46]. However, it is also important to understand the consequences of the addition of the large number of invasive species that do not displace native species [3]. In particular, when species displacements do not occur, it is important to examine the role of non-lethal, indirect effects of non-native species on ecosystem services.

In our study, the direct effect of the non-native *Petrolisthes* is extremely low because the crab likely does not eat phytoplankton, as we observed no chlorophyll *a* drawdown for this species. Caine [47] reports that *Petrolisthes* mostly consumes zooplankton and detritus; any algae that is ingested is apparently benthic in origin. Thus, the slightly positive effect of the crab on water column chlorophyll *a* concentrations may be explained by observations that *Petrolisthes* affects localized water currents, which could hinder phytoplankton from settling. In contrast, groups of oysters drew down substantial amounts of chlorophyll *a*, and clams a moderate, but significant amount, corroborating previous findings that *C. virginica* per capita feeding rates are typically higher than *Mercenaria* clams [18], [48]. Although clam effects on chlorophyll *a* were smaller, when combined with oysters, the two species had an additive effect. Such additivity, as opposed to competitive or inhibitory effects, was likely facilitated by the high resource levels available in this experiment.

In the simplest combinations of species there were no signs of an indirect trait-mediated effect of *Petrolisthes*. That is, in the absence of predators, *Petrolisthes* had no effect on clam and oyster net filtration, underscoring that this invasive crab is likely not in competition for resources with the filter-feeding bivalves. Even without resource competition, we had hypothesized that tactile disturbance from the crabs might cause periodic feeding cessation in the bivalves, but we did not observe any decreases in chlorophyll *a* drawdown rates by the bivalves when *Petrolisthes* was present.

However, the native mud crab predator did differentially influence filtration rates of the species. Oysters maintained the same filtration rate as when predators were absent. However, clams were very sensitive to predators and ceased filtration altogether. In the presence of predators, chlorophyll *a* drawdown in the oyster-clam treatment was equivalent to the oyster-only treatment, which is consistent with the notion that clam filtration was inhibited by predator presence. The indifference to mud crabs by oysters may be explained by large oyster sizes. Mud crabs generally prey on juvenile oysters, and the large adults we used in this study are largely an invulnerable size (e.g., [49], [50]). The clams' sizes in our study make them equally invulnerable to *P. herbstii* predation (e.g., [51], [52]), but they responded more sensitively to predators. This heightened sensitivity existed despite the fact that we seldom observed *P. herbstii* handling the large clams. In the presence of predators *Petrolisthes* arrested its physical activity and chlorophyll *a* decreased to exactly match the no-species controls. That is, in limiting *Petrolisthes* activity, predator presence eliminated the small positive effect that *Petrolisthes* had on chlorophyll *a* levels (relative to the controls). Perhaps when *Petrolisthes* cease their filtering activity and movements they are no longer altering water currents and re-suspending phytoplankton.

Despite *Petrolisthes*' limited direct effect on filtration, the addition of *Petrolisthes* to treatments in the predator trials had an interesting indirect effect. In the combined clam and *Petrolisthes* treatment, *Petrolisthes* presence buffered predator effects on clams, and chlorophyll *a* drawdown by the clams was no longer negligible, but rather back at positive levels equivalent to those that had been measured when predators were absent. There was also a trend of this facilitative buffering effect by *Petrolisthes* in the three-species treatment, but we did not observe this effect in the oyster and *Petrolisthes* combined treatment. Taken together, these results suggest that the clams were able to filter at their previously unadulterated rates even in the presence of a predator when *Petrolisthes* was also present. The mechanism involved here is uncertain, but abundant *Petrolisthes* may habituate and desensitize clams to tactile or olfactory crab stimuli, such that clams continue filtering in the presence of mud crab predators. Mud crabs also appear to target *Petrolisthes* more strongly than the adult clams, which is supported by our observation of multiple, dismembered dead *Petrolisthes* at the end of the predator trails, and by the known high prey value of *Petrolisthes* to *P. herbstii* [53]. There is no discernible buffering effect of *Petrolisthes* on the adult oysters, which were already indifferent to predatory crab presence.

In our controlled lab setting with a fixed numbers of predators and prey, the invasive *Petrolisthes* seemingly allows a more fully functioning ecosystem service of higher net water filtration by clams and thus the collective oyster reef community. However, it is uncertain if this positive influence will hold in natural field settings with dynamic populations. In particular, it should be cautioned that if *Petrolisthes* as a new food resource boosts *P. herbstii* populations, this could create attendant negative effects of apparent competition on the populations of native prey species [54], [55], which in the long run could diminish the prey's collective filtration services. Longer term studies that account for shifts in population equilibria due to *Petrolisthes*' introduction, as well as field trials that incorporate natural reef conditions, are clearly needed to complement the performance assessment we have conducted here on the relative impact of non-native species on the critical ecosystem service of water filtration.

In summary, this study shows the largest portion of oyster reef filtration is attributable to oysters, which have a naturally high filtration rate that is not compromised by native mud crab predators or the invasive *Petrolisthes*. Clam filtration is notably diminished by mud crab presence; however, chlorophyll *a* drawdown by clams may be restored in the presence of the common non-native crab *Petrolisthes. Petrolisthes* likely does not compete with bivalve filter feeders for similar foods and may facilitate clam filtration by desensitizing the clams to predator cues or reducing the predator's tactile perturbations that stem from its foraging and sediment probing. More generally, our study demonstrates that non-native species can have differential, non-lethal effects on native species that can alter ecosystem services. Although such trait-mediated indirect effects are more subtle to capture than direct effects on density, their important influence seen here, coupled with the increasing spread of non-native species across all types of ecosystems, suggests the compelling need to understand how commonly these kind of effects occur.
